# Anesthetic Management Using Epidural Analgesia for Emergency Laparoscopic Cholecystectomy in a Patient with Lupus Anticoagulant Positivity and Prolonged Activated Partial Thromboplastin Time

**DOI:** 10.1155/2022/6310630

**Published:** 2022-01-18

**Authors:** Yasuhiro Watanabe, Toru Kaneda

**Affiliations:** Department of Anesthesia, Japanese Red Cross Shizuoka Hospital, 8-2 Oute-machi Aoi-ku, Shizuoka 420-0853, Japan

## Abstract

Lupus anticoagulant (LA), an antiphospholipid antibody, prolongs *in vitro* activated partial thromboplastin time (APTT) despite the presence of a hypercoagulable state *in vivo*. Irrespective of whether they receive antithrombotic therapy, meticulous anesthetic management is imperative in patients with LA positivity to prevent thrombotic complication. Additionally, emergency surgery in such patients can be challenging, as the time to devise perioperative strategies is limited. Here, we described the case of a patient with LA positivity and prolonged APTT who underwent emergency laparoscopic cholecystectomy with successful anesthetic management using epidural analgesia. An 83-year-old woman presented with acute cholecystitis and underwent emergency laparoscopic cholecystectomy. Preoperative blood test results revealed a prolonged APTT of 83 s, prothrombin time/international normalized ratio of 1.14, and normal platelet count. The patient had experienced a marked prolongation of APTT ten years previously, which was attributed to LA positivity, and she had previously undergone surgery for rectal cancer under general and epidural anesthesia. The patient did not receive antithrombotic therapy, and she demonstrated neither liver dysfunction nor a bleeding tendency. We prioritized optimal analgesia to enable early mobilization; therefore, an epidural catheter was placed in preparation for transition to open abdominal surgery. The operation was completed under laparoscopy, and good pain control was achieved postoperatively with continuous epidural analgesia, facilitating early ambulation. The epidural catheter was removed on the second postoperative day, and the patient did not develop any signs of thromboembolism or neurologic complications during her hospital stay. Anesthetic management for emergency laparoscopic cholecystectomy was successfully performed using epidural analgesia in a patient with LA positivity and prolonged APTT. Careful evaluation of laboratory data, treatment history, and clinical symptoms is of critical importance in such patients.

## 1. Introduction

Antiphospholipid syndrome (APS) is an autoimmune disorder characterized by antiphospholipid antibody (aPL) positivity and clinical manifestations such as vascular thrombosis and pregnancy morbidity [1]. Lupus anticoagulant (LA), one of the diagnostic aPLs for APS [2], targets the epitopes of the negatively charged phospholipid-binding protein, resulting in prolongation of phospholipid-dependent *in vitro* coagulation, which is reflected in the results of tests such as activated partial thromboplastin time (APTT) [3]. However, LA positivity in itself is a risk factor for thrombosis, and a hypercoagulable state should be assumed in patients with LA positivity despite a prolonged or normal APTT. Irrespective of whether they receive antithrombotic therapy, meticulous anesthetic management is imperative in patients with LA positivity to prevent thrombotic complication. In addition, emergency surgery in such patients can be especially challenging, as the time to devise perioperative strategies is limited. Here, we described the case of a patient with LA positivity and prolonged APTT who underwent emergency laparoscopic cholecystectomy with successful anesthetic management using epidural analgesia.

## 2. Case Presentation

An 83-year-old woman (height, 152 cm; weight, 54 kg) was admitted to our hospital due to acute cholecystitis and underwent emergency laparoscopic cholecystectomy on the third day of hospitalization. The surgeons judged the probability of conversion to open cholecystectomy to be considerably high. The patient had been prescribed atorvastatin calcium trihydrate (5 mg) for treating hypercholesterolemia and had previously undergone hemithyroidectomy for thyroid cancer, venous graft bypass for a venous aneurysm, and rectal resection with colostomy for rectal cancer. Electrocardiography revealed sinus rhythm with a heart rate of 82 beats per minute, prolonged QT interval (QTc value, 0.478), and complete right bundle branch block. Chest radiographs showed clear lung fields, and transthoracic echocardiography revealed an ejection fraction of 72%, normal ventricular wall motion, moderate tricuspid valve regurgitation, and no intracardiac thrombi. The results of preoperative blood tests showed a markedly prolonged APTT of 83 s (reference value, 24–36 s), prothrombin time/international normalized ratio (PT-INR) of 1.14, white blood cell count of 15,620/*μ*L, hemoglobin (Hb) level of 12.2 g/dL, hematocrit (Hct) level of 37%, platelet count of 16.5 × 10^4^/*μ*L, C-reactive protein level of 8.8 mg/dL, free triiodothyronine level of 1.31 pg/mL, and thyroid stimulating hormone level of 0.43 *μ*IU/mL. The other results were unremarkable.

A review of the patient's medical records revealed that fourteen years previously, she had experienced venous graft occlusion after venous aneurysm resection despite alprostadil alfadex administration and unfractionated heparin infusion. Furthermore, an abnormal prolongation of APTT (fluctuated between 72 and 88 s) with a normal PT-INR (around 1.0) had first been identified at the time of preoperative examination for rectal cancer surgery 10 years previously. On hematological examination, LA positivity had been noted on three occasions within 1 month, and after a thorough investigation, the APTT prolongation had been attributed to LA positivity. Other aPLs, including anticardiolipin antibody (aCL) and anti-*β*2 glycoprotein I antibody (anti-*β*2GPI), had not been detected. Taking the thrombotic venous graft occlusion and LA positivity into consideration, the patient had clinically been diagnosed with APS and underwent surgery for rectal cancer under general and epidural anesthesia. Low-molecular-weight heparin (LMWH) had been administered postoperatively, and no neurological symptoms had appeared after the epidural catheter was removed. Thereafter, although the patient has not received antithrombotic therapy to date, she did not experience thromboembolism.

During the current admission, we did not fully attribute the abnormal results of coagulation tests, particularly the PT-INR value of 1.14, to LA positivity (i.e., coagulopathy due to inflammation). The patient demonstrated neither liver dysfunction nor signs of bleeding tendency such as subcutaneous petechiae. Furthermore, on chronologically comparing the APTT and PT-INR values, we did not consider the abnormal values to be contraindications to neuraxial blockade. Therefore, we decided to administer epidural analgesia for optimal postoperative analgesia and to enable early mobilization.

In the operating room, an epidural catheter was inserted at the T8-T9 intervertebral space using an 18-gauge Tuohy needle with the loss-of-resistance technique and fixed at 9 cm deep to the skin. General anesthesia was induced with 100 *μ*g fentanyl, 1 mg midazolam, 30 mg propofol, and 50 mg rocuronium, and standard monitoring was performed. After performing rapid sequential intubation, anesthesia was maintained with desflurane, continuous infusion of remifentanil (0.05–0.2 *μ*g/kg/min), and epidural administration of 0.2% ropivacaine. Metoclopramide hydrochloride (10 mg) was administered at the beginning of the surgery. Mechanical thromboprophylaxis with elastic stockings and intermittent pneumatic compression was performed perioperatively. Intraoperatively, patient-controlled epidural analgesia (PCEA) with 0.2% ropivacaine (basal, 4 mL; bolus, 2 mL; lock-out time, 30 min) was initiated, and blood pressure was maintained with several boluses of ephedrine and phenylephrine. Although the gallbladder was strongly adhered to adjacent tissues, cholecystectomy was completed under laparoscopy. The operation time and total anesthesia time were 322 min and 362 min, respectively. The amount of fluid administered intraoperatively was 3350 mL, including 280 mL of red blood cells, whereas the blood loss and urine output were 670 g and 100 mL, respectively. The patient was extubated in the operating room and transferred to the general ward. On the first postoperative day, blood test results showed an APTT of 70 s, PT-INR of 1.19, fibrin/fibrinogen degradation product level of 38 *μ*g/mL, Hb level of 11.3 g/dL, Hct level of 33%, and platelet count of 11.3 × 10^4^/*μ*L. There was no nausea, vomiting, or pain at rest, and the patient ambulated using PCEA with a single infusion of 1000 mg acetaminophen. On the second postoperative day, the epidural catheter was removed, and the patient developed no signs or symptoms of thromboembolism thereafter. Pathological examination of the specimen revealed gallbladder carcinoma, and 7 days postoperatively, the patient was discharged without any neurologic complications.

## 3. Discussion

We highlighted the significance of case-oriented anesthetic management in a patient with LA positivity. Our patient did not strictly meet the current criteria for APS, as LA positivity was not detected multiple times at least 12 weeks apart [2], and it is somewhat atypical that the patient has not received antithrombotic therapy, likely because of the marginal thrombotic event. Due to the retrospective nature of this report, we could not ascertain the reasons for these decisions; however, considering that aPLs are present in 1–5% of the general population [3], and some aPL carriers with no prior history of thrombotic event can be followed up without medication [4, 5], anesthesiologists might be involved in the anesthetic management of such patients.

In the present case, we could use epidural analgesia for the following reasons: first, a chronological comparison revealed that the APTT and PT-INR values did not deviate enough to preclude epidural catheter placement. Second, antithrombotic therapy has not been administered, and blood test results demonstrated normal liver function and platelet counts. Finally, there were no clinical symptoms of a bleeding tendency. Theoretically, neuraxial blockade, including epidural analgesia, can be performed in patients with APS and aPL positivity, as a hypercoagulable state is the essential pathophysiology in such patients [6]. Additionally, in some patients receiving antithrombotic therapy, meticulously performed bridging anticoagulation allows the use of regional anesthesia [7]. Nevertheless, these procedures tend to be avoided in both emergency and elective settings; therefore, we aimed to discuss the implications of using epidural analgesia in an emergency patient with aPL positivity.

Here, the emergency cholecystectomy was completed under laparoscopy. However, if conversion to open cholecystectomy would have been needed, continuous intravenous analgesia with narcotics would have been used, resulting in nausea, vomiting, and immobilization. The second-hit theory proposes that aPL-positive patient requires an initiating event such as infection, trauma, and prolonged immobilization, to develop thrombosis [8]; therefore, surgery itself can be a priming factor for thrombogenesis. In addition, malignancy, in the present case which was revealed by postoperative pathological examination, is an established risk factor for venous thromboembolism [9]. Although our patient did not have multiple aPL positivity which represents high-risk aPL profile [5], given that LA positivity is a stronger risk factor for thrombosis than positivity for other aPLs [10, 11], we prioritized optimal analgesia with epidural catheter placement to facilitate early mobilization. Droperidol was not administered as a part of PCEA because the patient had QT prolongation, and narcotics were also not loaded to avoid nausea and vomiting. In addition, a sufficient amount of fluid was administered to prevent postoperative hypotension, which might result in discontinuation of PCEA.

It has been recommended that aPL-positive patients, including those not receiving chronic antithrombotic therapy, are anticoagulated with unfractionated heparin or preferably LMWH in the perioperative period [12]. In the present case, postoperative LMWH was not administered because the PT-INR was prolonged to 1.19 on the first postoperative day, and the surgeons preferred avoiding hemorrhage from the intra-abdominal surgical site to providing aggressive thromboprophylaxis. The decision is not necessarily inappropriate, as the timing of anticoagulation therapy initiation can be determined by assessing postoperative hemostasis even in aPL-positive patients [6]. It has been reported that a significant anticoagulation effect is present at the time of epidural catheter removal in patients receiving twice-daily LMWH administration than in those receiving once-daily LMWH administration [13]. Hence, generally, twice-daily LMWH administration is not recommended in patients with an indwelling epidural catheter, while once-daily administration is also controversial due to lack of evidence regarding its efficacy and safety [14]. In cases where LMWH has been administered immediately after surgery, the epidural catheter should be removed at least 12 h after administration with confirmation through coagulation tests [14].

In conclusion, epidural analgesia was used for optimal anesthetic management without thrombotic and hemorrhagic complications in an emergency patient with LA positivity and prolonged APTT. Careful evaluation of laboratory data, treatment history, and clinical symptoms is critical in such patients.

## Data Availability

All relevant data are included in this article. Other datasets are available from the corresponding author upon request.
